# Effects of concurrent neuropathologies with Alzheimer disease neuropathologic change on cognitive decline: Minimal impact of vascular brain injury compared with other combinations

**DOI:** 10.1093/jnen/nlag033

**Published:** 2026-04-23

**Authors:** Sarah M Yasuda, Charles Mock, Kathryn Gauthreaux, Kwun C G Chan, C Dirk Keene, Jessica E Culhane, Yen-Chi Chen, Walter Kukull

**Affiliations:** Department of Epidemiology, National Alzheimer’s Coordinating Center, University of Washington, Seattle, WA, United States; Department of Epidemiology, National Alzheimer’s Coordinating Center, University of Washington, Seattle, WA, United States; Department of Epidemiology, National Alzheimer’s Coordinating Center, University of Washington, Seattle, WA, United States; Department of Epidemiology, National Alzheimer’s Coordinating Center, University of Washington, Seattle, WA, United States; Department of Biostatistics, University of Washington, Seattle, WA, United States; Department of Laboratory Medicine and Pathology, University of Washington, Seattle, WA, United States; Department of Epidemiology, National Alzheimer’s Coordinating Center, University of Washington, Seattle, WA, United States; Department of Epidemiology, National Alzheimer’s Coordinating Center, University of Washington, Seattle, WA, United States; Department of Statistics, University of Washington, Seattle, WA, United States; Department of Neurology, Washington University School of Medicine, St. Louis, MO, United States

**Keywords:** Alzheimer disease, Lewy body, limbic predominant age-related TDP-43 encephalopathy, multiple neuropathology, vascular brain injury

## Abstract

We examined cognitive changes associated with several neuropathologic entities, alone and in combination. We studied 808 participants from the National Alzheimer’s Coordinating Center to assess associations between neuropathologic diagnoses (from autopsy) and neuropsychologic test scores (trajectories over time for 5 domains: overall cognition, episodic memory, attention, language, executive function). Neuropathologies included: Alzheimer disease neuropathologic change (ADNC), Lewy body disease (LBD), vascular brain injury (VBI), and limbic-predominant age-related TDP43 encephalopathy neuropathologic change (LATE-NC). Using linear mixed-effects models, we examined trajectories of cognitive decline for ADNC alone compared to ADNC plus LBD, VBI, or LATE-NC. We also examined differences between observed trajectories and trajectories that would be expected if the neuropathologic entities exerted their effects independently (additively). ADNC+LBD had worse decline than ADNC alone for 4 of the 5 domains with rate of decline consistent with an additive model for all 4 domains. ADNC+LATE-NC had worse decline than ADNC alone for 3 domains with rate of decline additive for only one and <additive for 2. ADNC+VBI had worse decline than ADNC alone for only one domain, with rate of decline <additive. These findings are relevant for prognostication in clinical practice and for clinical trial design.

## Introduction

Dementia is characterized by a range of cognitive symptoms such as impaired ability to recall, reason, or make decisions. It disrupts daily activities for millions of Americans. The number of people affected is expected to reach 14 million by 2060.^1^ There are a wide range of neuropathologic processes that can cause dementia; we focus on the most prevalent sporadic causes of dementia, including Alzheimer disease neuropathologic change (ADNC). In the United States, ADNC has been found in 60 to 80% of people with dementia.^2^^,^^3^ Other neuropathologic entities that have been found to be strongly and broadly associated with dementia include: limbic-predominant age-related TDP-43 encephalopathy neuropathologic change (LATE-NC), Lewy body disease (LBD), and vascular brain injury (VBI).

Although ADNC itself is common, in the community it is unusual to find this neuropathology in isolation. Rather, combinations of age-related neurodegenerative neuropathologies are more common. An autopsy study by Boyle et al. found that among 1079 participants, 78% had evidence of 2 or more neuropathologic entities, 58% had 3 or more, and only 9% had isolated ADNC.^4^ Furthermore, their study identified 236 unique combinations of neuropathologic entities. Among their participants, 43% were diagnosed with Alzheimer disease (ad) dementia and 25% were diagnosed with mild cognitive impairment (MCI) at the time of their death.^4^ Similarly, a study on 571 brain donors who had been exposed to repetitive head impacts found 252 unique combinations of neuropathologic entities.^5^ Approximately half (53%) of the participants in that study had dementia at the time of their death.^5^ These findings display the heterogeneity and the complexity of neuropathology in aging and highlight the need for a comprehensive understanding of the effects of combinations of multiple neuropathologic entities.

As expected, a higher number of neuropathologic entities has been associated with worsened cognitive performance.^2^^,^^6^^,^^7^ Many studies have examined the cognitive effects of combinations of 2 neuropathologic entities, usually in comparison to the cognitive effects of one of the neuropathologic entities alone.^8–16^ For example, Gauthreaux et al. showed that participants with ADNC and LATE-NC had worse Clinical Dementia Rating (CDR) global scores (from the CDR Dementia Staging Instrument) than those with ADNC alone.^8^ Similar effects are also seen with other neuropathologic combinations such as ADNC plus LBD and ADNC plus microinfarcts.^9^^,^^10^

Studies examining the effects of and interactions among multiple neuropathologic entities have been less common. A study by Brenowitz et al. examined the effect of mixed neuropathologic entities (ADNC, VBI, and LBD) on longitudinal cognitive decline using the National Alzheimer’s Coordinating Center (NACC) dataset. Participants with 2 neuropathologic entities had worsened cognitive decline vs participants with 1 neuropathologic entity. However, it was notable that mixed neuropathologies had < additive effects on cognitive decline.^3^ For example, participants with both ADNC and LBD had a decline in episodic memory that was slower than would have been expected if the 2 neuropathologic entities exerted their effects in an additive fashion. In a different autopsy series, Schneider et al. also showed that ADNC and LBD had an additive but not synergistic (ie, worse than additive) effect on the probability of dementia.^17^ Similarly, in an in vivo study using imaging to assess the presence of vascular and amyloid neuropathologies, Vemuri et al. showed that these 2 entities had an additive but not synergistic effect on a global cognitive summary score based on 9 neuropsychological tests.^16^

The current study sought to build on the above-noted studies and extend understanding of the effects of and interactions among multiple neuropathologic entities. We specifically sought to build on the foundation laid by Brenowitz et al. by adding LATE-NC to the list of neuropathologic entities analyzed. Although TDP-43 was known at the time of the study by Brenowitz et al., neither LATE-NC nor its staging had yet been defined.^3^^,^^18^ The current study also sought to extend the Brenowitz study by using a current version of the NACC dataset.

## Methods

### Participants and data sources

This study was conducted using the Uniform Data Set (UDS) from NACC to investigate the cognitive decline in overall cognitive performance and in 4 neuropsychological domains associated with each individual neuropathologic entity and with several combinations of neuropathologic entities. The UDS contains longitudinal data from 2005 onward from over 40 Alzheimer’s Disease Research Centers (ADRCs) funded by the National Institute on Aging (NIA) across the United States.^19–23^ The UDS represents over 54000 participants with cognitive status ranging from normal cognition to mild cognitive impairment (MCI) to dementia. Participants were recruited in accordance with the protocols established by each ADRC, including clinical referral, self-referral, and active recruitment in community organizations. In many centers, recruitment extends to volunteers with normal cognition. Data from participants and co-participants (ie, a friend or family member), were collected by trained clinicians during in-person office visits, home visits, or telephone calls.

Data on neuropathologic entities present at the time of death are available for UDS participants who consented to autopsy. Data on autopsy-related variables for this study were obtained from the NACC Neuropathology (NP) Data Set, which was collected using a standardized neuropathological evaluation. Versions 10 to 11 of the NP dataset include the 2012 NIA-AA criteria for Alzheimer’s disease as well as more detailed information on vascular, TDP-43, and other neuropathologic entities.^24^ A neuropsychological battery was administered at each UDS visit (either NACC Form C1 or C2). The C2 form was implemented in 2015 to replace C1 tests that were used under licensing restrictions.

ADRCs obtained written informed consent from their participants and have their own separate IRB review and approval from their institution prior to submitting data to NACC.

### Inclusion and exclusion criteria

Participants who were deceased, were 65 or older at the time of death, and had undergone neuropathologic evaluation using the NP form version 10 or later were included in the study, as data on TDP-43 were not collected prior to version 10, which was implemented in 2014. Participants with rare pathologies (such as Down syndrome, pigment-spheroid degeneration, multiple system atrophy, prion disease, trinucleotide disease, malformation of cortical development, metabolic storage disorder of any type), white matter disease (leukodystrophy, multiple sclerosis or other demyelinating disease), acute or chronic contusion/traumatic brain injury of any type, primary or metastatic neoplasm, encephalitis, abscess, or other infectious process of any type, herniation, ALS/motor neuron disease, cerebral autosomal dominant arteriopathy with subcortical infarcts and leukoencephalopathy (CADASIL), frontotemporal lobar degeneration (FTLD) with tau, FTLD with TDP43, and other FTLD were excluded from this study. Participants who were not assessed for any of the following neuropathologic entities were excluded from the study: ADNC, old infarcts and old microinfarcts in the cerebral cortex, subcortical or periventricular white matter, and subcortical gray matter, TDP-43 neuropathology in the hippocampus or neocortex, or Lewy body disease. Participants whose last visit occurred over 2 years before their death were excluded from the study to capture a more accurate representation of the association between the participant’s cognitive condition and the neuropathology. In order to examine longitudinal changes in cognition, participants with < 2 clinical visits were excluded. Participants who met study inclusion criteria were selected from the March 2025 data freeze.

### Cognitive performance

Cognitive performance was measured using scores from the UDS neuropsychological battery. Each participant had a set of either C1 or C2 test scores for each visit. Neuropsychological battery scores were grouped into 4 cognitive domains as previously established by Hayden et al.^25^ episodic memory (immediate and delayed WMS-R logical memory for C1 or immediate and delayed Craft story 21 recall for C2), attention/working memory (forward and backward digit span for C1 or forward and backward number span for C2), semantic memory/language (category fluency of animals and vegetables and either Boston naming test for C1 or multilingual naming test [MINT] for C2), and executive function (trail making test part A and B). Participants also had an overall cognitive performance score: either the Mini-Mental State Examination ([MMSE], for C1) or the Montreal Cognitive Assessment ([MoCA], for C2). Z-scores were derived for each of the test scores, using the mean and standard deviation of that test score for all initial visit scores among cognitively normal participants (global CDR = 0) in the UDS. Z-scores for participants in the current study were then calculated by subtracting their score from the mean score for the cognitively normal participants and dividing the difference by the standard deviation.

### Neuropathologic entities

Neuropathologic entities including ADNC, LBD, VBI, and LATE-NC were analyzed. Restrictive definitions of the 4 diagnostic categories were used to reflect moderate to severe neuropathology, which is generally considered to be sufficient explanation for cognitive impairment for each of the specific neuropathologies.^26^ ADNC was defined as having intermediate or high ADNC based on the National Institute on Aging and Alzheimer’s Association (NIA-AA) ADNC ABC score. ABC score is based on (1) Aβ/amyloid plaque distribution, (2) neurofibrillary tangle distribution (Braak stage), and (3) neuritic plaque density in the neocortex (CERAD).^26^ LBD was defined by the presence of neocortical Lewy bodies. VBI was defined by any gross infarct and/or 2 or more microinfarcts in any of the following 3 regions: cortical, subcortical (or periventricular) white matter, or subcortical deep gray matter. LATE-NC/TDP-43 was defined by the distribution of TDP-43 immunoreactive inclusions in hippocampus or neocortex (ie, LATE-NC stages 2 or 3). Using these 4 neuropathologic entities, 15 different combinations were created. However, due to small sample size in categories without ADNC and with 3 or more neuropathologic entities, cognitive changes were analyzed for only 7 categories (ADNC-only, LBD-only, VBI-only, LATE-NC-only, ADNC + LBD, ADNC + VBI, and ADNC + LATE-NC) along with a “no neuropathologic entity” (No NP) category for participants with no evidence of the 4 main entities.

### Statistical analysis

Descriptive statistics were conducted to analyze participant characteristics for each neuropathologic combination. These characteristics included: age, sex, education (<16 years vs ≥16 years [college graduate]), ethnicity/race (white vs non-white), history of stroke (absent vs present), *APOE* E4 allele (present vs absent), cognitive status at the last visit (normal cognition, impaired no MCI, MCI, or dementia), CDR sum of boxes (SB) at the first and last visit, Braak stage (0 to 4 vs 5 to 6), hippocampal sclerosis (none vs unilateral, bilateral, and present but laterality not assessed), severe arteriolosclerosis (none, mild, and moderate vs severe), severe atherosclerosis (none, mild, and moderate vs severe), cerebral amyloid angiopathy (none vs mild, moderate, and severe), gross infarct (0 vs ≥1), and micro infarcts (0, 1, 2, or ≥3).

A linear mixed-effects model was used to estimate the annual change in the cognitive domain z-scores for each neuropathologic category. Categorical variables for each of the 8 neuropathologic entities or combinations were used as independent variables. Random intercepts for ADRC and unique participant ID were included in the model to account for the individual and center-level variations. If there was a missing test score, the respective domain score was coded as missing. The same method was used to calculate the overall cognitive performance. The model adjusted for sex, education, race, age at death, neuropsychological battery form version (C1 or C2), and global CDR score at baseline.

The same linear mixed-effects models were used to estimate the differences in annual change in the cognitive domain z-scores for ADNC compared with combinations of ADNC with other neuropathologic entities (ie, ADNC + LBD; ADNC + LATE-NC; and ADNC + VBI). Similarly, these linear mixed-effects models were used to estimate the differences in annual change in the cognitive domain z-scores for the observed slopes compared with what would have been expected in an additive model, in which each neuropathologic entity exerted an independent effect on cognition. The observed annual change (slope) for each pathology group was calculated by adding the fixed-effect coefficient of time representing the annual change, and the time and pathology group interaction coefficient. The additive slopes were the sum of 2 observed slopes corresponding to single-pathology groups (eg, “ADNC Only” and “LBD Only”). The difference between the observed and additive slopes for each combined pathology, taking the ADNC + LBD group as an example, was calculated as β_ADNC+LBD×time_−(β_time_+β_ADNC Only×time_+β_LBD Only×time_). This observed vs additive model was compared for each of the above noted combinations (ie, ADNC + LBD; ADNC + LATE-NC; and ADNC + VBI). Interactions between 2 neuropathologies were considered as additive (consistent with an additive model) if the cognitive decline associated with the combined neuropathologies was equivalent to the sum of their respective individual declines. Interactions were considered synergistic if they were significantly worse (worse cognitive decline) than in an additive model. Interactions were considered antagonistic if they were significantly better (less cognitive decline) than in an additive model.

### Sensitivity analysis

As noted above, for the main analysis we used restrictive definitions of the 4 diagnostic entities. We also conducted 2 sensitivity analyses using broader neuropathologic definitions. For the first sensitivity analysis, the definitions were ADNC (low, intermediate or high on the NIA-AA ABC score), LBD (Lewy bodies in any brain region), VBI (any gross infract or any microinfarct in any of the following 4 regions: cortical, subcortical [or periventricular] white matter, subcortical deep gray matter, or brainstem/cerebellum), and LATE-NC (TDP-43 in any of: amygdala, hippocampus, neocortex). For the second sensitivity analysis, the same definitions were used except that the definition of VBI included moderate to severe atherosclerosis (in the circle of Willis) or moderate to severe arteriolosclerosis (in the subcortical white or gray matter). For both sensitivity analyses, the same statistical methods were used as for the main analysis. The rationale for including microinfarcts in the brainstem/cerebellum and including atherosclerosis and arteriolosclerosis is that although these findings would not cause cognitive changes in-and-of themselves, they are markers for more severe cerebrovascular disease and are potentially reflective of vascular brain injury not detected at autopsy.

A final exploratory analysis was performed in which we assessed the significance of differences in the 5 cognitive domains for the individual neuropathologies (LBD, LATE-NC, VBI) alone versus in combination with ADNC (LBD + ADNC; VBI + ADNC; LATE-NC + ADNC). This was done using the same neuropathologic definitions and the same statistical methods as in the main analysis. Note: this analysis was similar to the main analysis, but compared the effects of ADNC + other neuropathologies to the effect of those individual neuropathologies alone, rather than to ADNC alone, as in the main analysis.

## RESULTS

**Figure 1:**
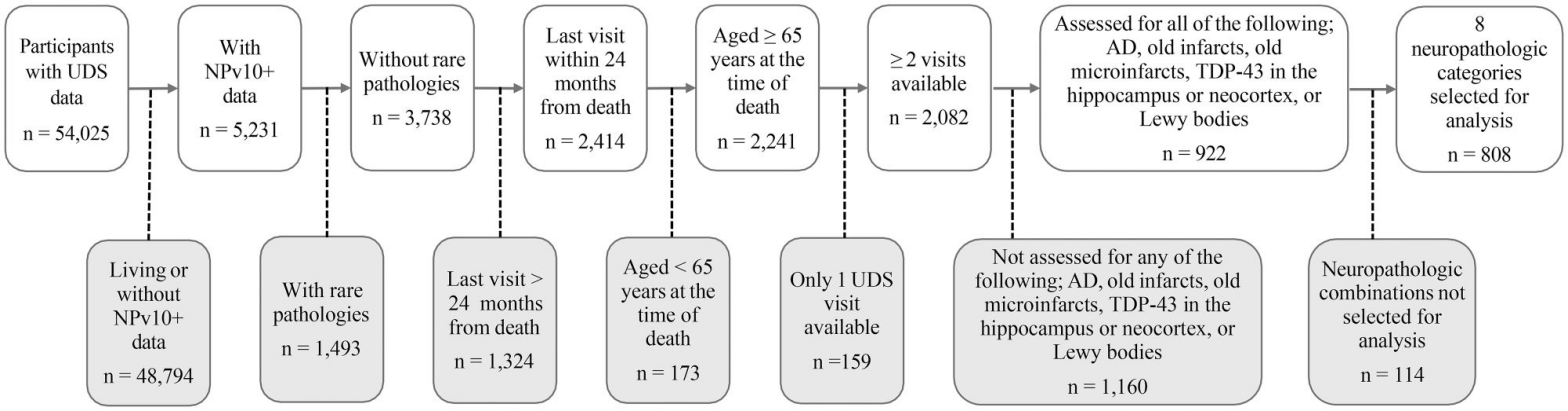
Sample inclusion and exclusion criteria.

**Figure 2:**
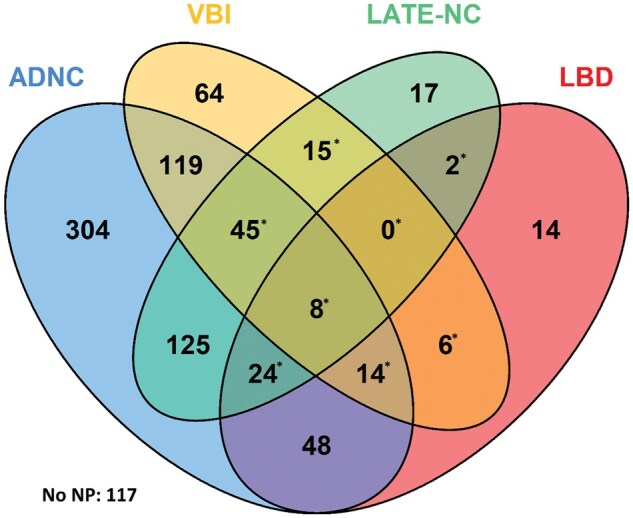
Distribution of selected neuropathologic entities. Sample size for all combinations: 922. *Excluded from the analysis for cognitive changes. Sample size for analytic sample for cognitive changes: 808. Abbreviations: ADNC = Alzheimer disease neuropathologic change; LATE-NC = limbic predominant, age related, TDP-43 encephalopathy; LBD = Lewy body disease; NP = neuropathology; VBI = vascular brain injury. Neuropathology definitions: ADNC = intermediate or high NIA-AA Alzheimer’s disease neuropathologic change (ADNC) (ABC score); LBD = Lewy body in Neocortical (diffuse) region; VBI = any gross infarct in the cerebral cortex, subcortical or periventricular white matter, or subcortical gray matter; and/or two or more microinfarcts in the cerebral cortex, subcortical or periventricular white matter, or subcortical gray matter; LATE/TDP-43 = distribution of TDP-43 immunoreactive inclusions in hippocampus or neocortex.

There were 922 participants with data on the 4 neuropathologic entities included in this study (Figure 1). Among these 922 participants, 74.5% (n = 687) had ADNC, 29.4% (n = 271) had VBI, 25.6% (n = 236) had LATE-NC, 12.6% (n = 116) had LBD, and 12.7% (n = 117) had none of these neuropathologies (Figure 2). Furthermore, 44.0% (n = 406) of participants had more than 1 neuropathologic entity, the most common of which were ADNC + LATE-NC (n = 125) and ADNC + VBI (n = 119). The following is the breakdown of participants who had only one neuropathology: ADNC (33.0%, n = 304), VBI (7.0%, n = 64), LATE-NC (1.8%, n = 17), and LBD (1.5%, n = 14). Following the exclusion of neuropathologic combinations not included in the analysis for cognitive decline (LBD + VBI, VBI + LATE-NC, LBD + LATE-NC, and categories with 3 or more entities), the analytic sample for the calculation of cognitive trajectories (slopes) included 808 participants (Figures 1 and 2).

As shown in Table 1, in the analytic sample for calculation of cognitive trajectories, the average age at death was 83.7 years and most participants were white and had a college degree. The overall average CDR sum of boxes (SB) was 2.9 at the initial visit and 9.4 at the last visit. The most common cognitive status at the last visit was dementia (69.9%). Prevalence of dementia ranged from 23.1% in the no NP group to 93.8% in the ADNC + LBD group. Table 2 delineates the neuropathologic characteristics of the analytic sample. Most participants (55.0%) had Braak stage V or VI, which ranged from 0.9% in the no NP group to 81.6% in the ADNC + LATE-NC group. Most participants (62.4%) had cerebral amyloid angiopathy, which had not been used in the definition of any of the neuropathologic categories. All categories had some participants with cerebral amyloid angiopathy, including the no NP group.

**Table 1:** Clinical characteristics of participants with autopsy data stratified by ADNC, LBD, VBI, and LATE-NC neuropathologic entities.

n (%)	Overall	No NP	ADNC only	LBD only	VBI only	LATE-NC only	ADNC + LBD	ADNC + VBI	ADNC + LATE-NC
**Autopsy**	808	117	304	14	64	17	48	119	125
**Age at death (years), mean (SD)**	83.7 (8.9)	85.9 (8.4)	81.5 (9.0)	79.7 (9.7)	85.2 (8.5)	90.4 (9.9)	79.6 (7.4)	85.2 (8.2)	86.0 (8.5)
**Female**	385 (47.6)	55 (47.0)	150 (49.3)	2 (14.3)	30 (46.9)	10 (58.8)	18 (37.5)	59 (49.6)	61 (48.8)
**College graduate**	519 (64.6)	78 (67.2)	198 (65.3)	10 (71.4)	36 (58.1)	12 (70.6)	33 (68.8)	60 (50.4)	92 (74.2)
**Non-White**	65 (8.1)	9 (7.7)	21 (6.9)	0 (0.0)	13 (20.6)	1 (5.9)	4 (8.3)	8 (6.8)	9 (7.3)
**History of stroke**	32 (14.0)	2 (5.9)	7 (7.4)	0 (0.0)	5 (41.7)	0 (0.0)	1 (9.1)	9 (47.4)	3 (10.0)
**APOE e4 allele**	343 (46.5)	18 (16.7)	156 (57.6)	5 (35.7)	7 (12.7)	7 (43.8)	27 (65.9)	56 (48.3)	67 (57.3)
**CDR-SB at first visit, mean (SD)**	2.9 (3.8)	0.9 (2.4)	3.4 (3.8)	2.4 (1.8)	2.0 (3.5)	0.9 (1.9)	4.6 (4.5)	3.2 (4.3)	3.8 (3.9)
**CDR at last visit, mean (SD)**	9.4 (7.0)	2.5 (4.6)	10.4 (7.0)	9.1 (5.4)	6.3 (6.6)	6.3 (5.6)	12.8 (5.6)	10.4 (6.2)	12.9 (5.6)
**Cognitive status at the last visit**									
**Normal cognition**	135 (16.7)	65 (55.6)	32 (10.5)	1 (7.1)	18 (28.1)	4 (23.5)	1 (2.1)	9 (7.6)	5 (4.0)
**Impaired-not-MCI**	20 (2.5)	8 (6.8)	8 (2.6)	0 (0.0)	0 (0.0)	1 (5.9)	0 (0.0)	2 (1.7)	1 (0.8)
**MCI**	88 (10.9)	17 (14.5)	37 (12.2)	0 (0.0)	11 (17.2)	1 (5.9)	2 (4.2)	12 (10.1)	8 (6.4)
**Dementia**	565 (69.9)	27 (23.1)	227 (74.7)	13 (92.9)	35 (54.7)	11 (64.7)	45 (93.8)	96 (80.7)	111 (88.8)

Missing values (n, %): education (5, <1%), race (3, <1%), history of stroke (580, 72%), APOE (70, 9%).

Abbreviations: ADNC = Alzheimer disease neuropathologic change; CDR-SB = Clinical Dementia Rating Sum of Boxes; LATE-NC = limbic predominant, age related, TDP-43 encephalopathy neuropathologic change; LBD = Lewy body disease; NP = neuropathology; VBI = vascular brain injury.

Neuropathology definitions: ADNC = intermediate or high NIA-AA Alzheimer’s disease neuropathologic change (ADNC) (ABC score); LBD = Lewy body in Neocortical (diffuse) region; VBI = any gross infarct in the cerebral cortex, subcortical or periventricular white matter, or subcortical gray matter; and/or 2 or more microinfarcts in the cerebral cortex, subcortical or periventricular white matter, or subcortical gray matter; LATE/TDP-43 = distribution of TDP-43 immunoreactive inclusions in hippocampus or neocortex.

**Table 2:** Pathologic characteristics of participants with autopsy data stratified by ADNC, LBD, VBI, and LATE-NC neuropathologic entities.

n (%)	Overall	No NP	ADNC only	LBD only	VBI only	LATE-NC only	ADNC + LBD	ADNC + VBI	ADNC + LATE-NC
**Autopsy**	808	117	304	14	64	17	48	119	125
**Braak stage V and VI**	444 (55.0)	1 (0.9)	225 (74.0)	1 (7.1)	1 (1.6)	0 (0.0)	37 (77.1)	77 (64.7)	102 (81.6)
**Hippocampal sclerosis**	99 (12.3)	3 (2.6)	17 (5.6)	0 (0.0)	6 (9.4)	11 (64.7)	1 (2.1)	8 (6.8)	53 (42.4)
**Severe arteriolosclerosis**	113 (14.1)	4 (3.4)	45 (14.9)	1 (7.1)	11 (17.2)	0 (0.0)	3 (6.2)	31 (26.3)	18 (14.9)
**Severe atherosclerosis**	157 (19.6)	18 (15.5)	52 (17.3)	2 (14.3)	16 (25.0)	4 (23.5)	5 (10.4)	35 (29.7)	25 (20.0)
**Cerebral amyloid angiopathy**	504 (62.4)	36 (30.8)	212 (69.7)	2 (14.3)	21 (32.8)	4 (23.5)	34 (70.8)	98 (82.4)	97 (77.6)
**Gross infarct**	124 (15.3)	0 (0.0)	0 (0.0)	0 (0.0)	43 (67.2)	0 (0.0)	0 (0.0)	81 (68.1)	0 (0.0)
**Microinfarcts**									
**0**	607 (75.1)	102 (87.2)	274 (90.1)	12 (85.7)	22 (34.4)	13 (76.5)	41 (85.4)	42 (35.3)	101 (80.8)
**1**	100 (12.4)	15 (12.8)	30 (9.9)	2 (14.3)	4 (6.2)	4 (23.5)	7 (14.6)	14 (11.8)	24 (19.2)
**2**	41 (5.1)	0 (0.0)	0 (0.0)	0 (0.0)	16 (25.0)	0 (0.0)	0 (0.0)	25 (21.0)	0 (0.0)
**3+**	60 (7.4)	0 (0.0)	0 (0.0)	0 (0.0)	22 (34.4)	0 (0.0)	0 (0.0)	38 (31.9)	0 (0.0)

Missing values (n, %): arteriosclerosis (6, <1%), atherosclerosis (5, <1%).

Abbreviations: ADNC = Alzheimer disease neuropathologic change; LATE-NC = limbic predominant, age related, TDP-43 encephalopathy neuropathologic change; LBD = Lewy body disease; NP = neuropathology; VBI = vascular brain injury.

Neuropathology definitions: ADNC = intermediate or high NIA-AA Alzheimer’s disease neuropathologic change (ADNC) (ABC score); LBD = Lewy body in Neocortical (diffuse) region; VBI = any gross infarct in the cerebral cortex, subcortical or periventricular white matter, or subcortical gray matter; and/or 2 or more microinfarcts in the cerebral cortex, subcortical or periventricular white matter, or subcortical gray matter; LATE/TDP-43 = distribution of TDP-43 immunoreactive inclusions in hippocampus or neocortex.

**Figure 3:**
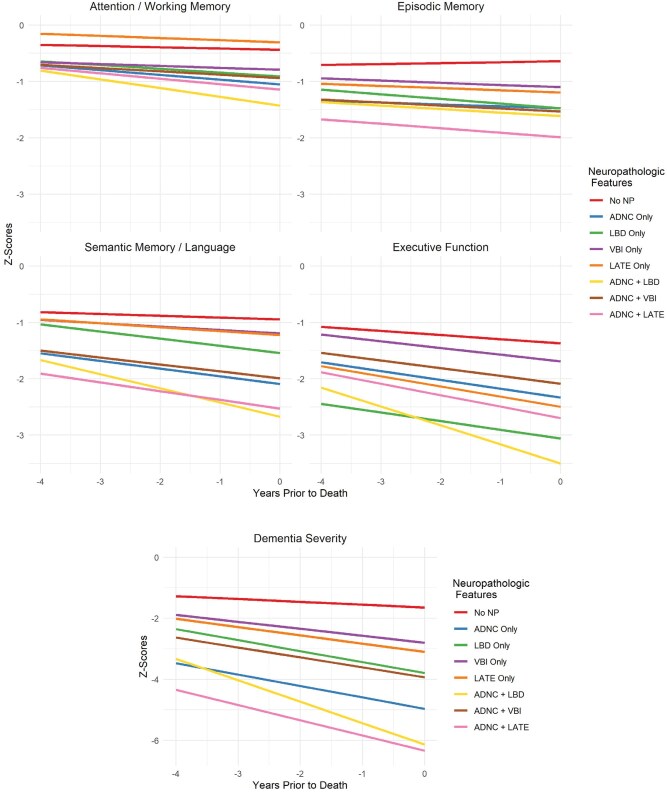
Model-based mean trajectories of cognitive domain z scores.

As shown in the model-based mean trajectories of cognitive performance of the 8 neuropathologic categories, participants with ADNC + LATE-NC or with ADNC + LBD had the lowest z-scores (worse performance) across most of the domains (Figure 3). Participants with no NP had the highest z-scores (best performance) for most of the measures (episodic memory, attention, language, executive function). Z-scores for attention and for episodic memory were more tightly clustered, with greater heterogeneity across the neuropathologic categories for overall cognition (dementia severity), language, and executive function.

All categories of neuropathology (including no NP) showed significant decline for all domains, except for episodic memory for the no NP group (Table 3). Rate of decline was fastest for ADNC + LBD and ADNC + LATE-NC for overall cognition, attention, language, and executive function, with lesser variations among the neuropathologic categories for episodic memory.

**Table 3:** Annual change in cognitive domain z scores by neuropathologic entities.

		Estimate (95% CI)		
Cognitive domains	Neuropathologic entities	Annual change in Z score	Difference between ADNC only and combinations	Difference between additive and observed slopes
**Overall cognitive performance (MMSE or MoCA)**	No NP	−0.09 (−0.14, -0.05)		
	ADNC Only	−0.37 (−0.41, -0.34)		
	LBD Only	−0.36 (−0.50, -0.22)		
	VBI Only	−0.23 (−0.29, -0.17)		
	LATE-NC Only	−0.27 (−0.39, -0.15)		
	ADNC + LBD	−0.70 (−0.82, -0.59)	**−0.33 (−0.45, -0.21)**	0.03 (−0.15, 0.22)
	ADNC + VBI	−0.33 (−0.38, -0.27)	0.05 (−0.01, 0.11)	**0.28 (0.19, 0.36)**
	ADNC + LATE-NC	−0.50 (−0.55, -0.44)	**−0.12 (−0.19, -0.06)**	**0.15 (0.02, 0.28)**
**Episodic memory**	No NP	0.02 (0.00, 0.03)		
	ADNC Only	−0.03 (−0.05, -0.02)		
	LBD Only	−0.08 (−0.13, -0.03)		
	VBI Only	−0.04 (−0.06, -0.02)		
	LATE-NC Only	−0.04 (−0.08, -0.00)		
	ADNC + LBD	−0.06 (−0.10, -0.02)	−0.03 (−0.07, 0.01)	0.05 (−0.01, 0.12)
	ADNC + VBI	−0.05 (−0.07, -0.04)	**−0.02 (−0.04, -0.00)**	0.02 (−0.01, 0.05)
	ADNC + LATE-NC	−0.08 (−0.10, -0.06)	**−0.05 (−0.07, -0.03)**	−0.01 (−0.05, 0.03)
**Attention/working memory**	No NP	−0.02 (−0.04, -0.01)		
	ADNC Only	−0.08 (−0.10, -0.07)		
	LBD Only	−0.07 (−0.11, -0.02)		
	VBI Only	−0.03 (−0.05, -0.01)		
	LATE-NC Only	−0.04 (−0.07, -0.00)		
	ADNC + LBD	−0.15 (−0.19, -0.12)	**−0.07 (−0.11, -0.03)**	−0.00 (−0.06, 0.06)
	ADNC + VBI	−0.06 (−0.07, -0.05)	**0.03 (0.01, 0.04)**	**0.06 (0.03, 0.08)**
	ADNC + LATE-NC	−0.10 (−0.11, -0.08)	−0.01 (−0.03, 0.01)	0.03 (−0.01, 0.06)
**Semantic memory/language**	No NP	−0.03 (−0.05, -0.02)		
	ADNC Only	−0.14 (−0.15, -0.12)		
	LBD Only	−0.13 (−0.18, -0.08)		
	VBI Only	−0.06 (−0.08, -0.04)		
	LATE-NC Only	−0.07 (−0.10, -0.03)		
	ADNC + LBD	−0.25 (−0.29, -0.21)	**−0.12 (−0.16, -0.08)**	0.01 (−0.05, 0.07)
	ADNC + VBI	−0.12 (−0.14, -0.11)	0.01 (−0.01, 0.03)	**0.07 (0.05, 0.10)**
	ADNC + LATE-NC	−0.16 (−0.17, -0.14)	−0.02 (−0.04, 0.00)	**0.05 (0.01, 0.09)**
**Executive function**	No NP	−0.07 (−0.09, -0.05)		
	ADNC Only	−0.16 (−0.17, -0.14)		
	LBD Only	−0.15 (−0.24, -0.07)		
	VBI Only	−0.12 (−0.15, -0.09)		
	LATE-NC Only	−0.18 (−0.23, -0.13)		
	ADNC + LBD	−0.34 (−0.39, -0.28)	**−0.18 (−0.24, -0.12)**	−0.03 (−0.13, 0.08)
	ADNC + VBI	−0.14 (−0.16, -0.11)	0.02 (−0.01, 0.05)	**0.14 (0.10, 0.18)**
	ADNC + LATE-NC	−0.20 (−0.23, -0.18)	**−0.05 (−0.08, -0.02)**	**0.13 (0.07, 0.19)**

Abbreviations: ADNC = Alzheimer disease neuropathologic change; LATE-NC = limbic predominant, age related, TDP-43 encephalopathy; LBD = Lewy body disease; NP = neuropathology; VBI = vascular brain injury. Each model was adjusted for sex, education, age at death, race, neuropsychological battery form version (C1 or C2), and global CDR score at baseline. Bold text indicates statistically significant.

ADNC + LBD had significantly worse decline than ADNC alone for 4 of the 5 domains (overall cognition, attention, language, executive function). For all of these 4 domains, the rate of decline was consistent with an additive model (ie, no significant differences between additive and observed slopes). ADNC + LATE-NC had significantly worse decline than ADNC alone for 3 of the 5 domains (overall cognition, episodic memory, executive function). For these 3 domains, the rate of decline was consistent with an additive model for 1 (episodic memory) and was < additive for 2 (overall cognitive performance and executive function). For these 2 domains there was a significant positive difference between additive and observed slopes, indicating less decline than anticipated with an additive model (ie, an antagonistic interaction). There was only one domain (episodic memory) for which ADNC + VBI had significantly worse decline than ADNC alone. For this domain, the rate of decline was significantly < anticipated from an additive model (ie, an antagonistic interaction). There was one domain (attention) for which ADNC + VBI had significantly less decline than ADNC alone. No interactions between any 2 neuropathologic entities were commensurate with a synergistic (worse than additive) effect.

### Sensitivity analysis

For the first sensitivity analysis (using broader definitions of the neuropathologic entities), the sample size for all participants decreased from 922 to 807 due to a high number of missing values for TDP-43 in the amygdala (n = 111) and for VBI in the brainstem/cerebellum (n = 4), neither of which had been used in the definition of the neuropathologic entities in the main analysis. Likewise, there was an increase in the number of people who had multiple neuropathologic combinations not included in the neuropsychological analysis, from 114 in the main analysis to 282 in the sensitivity analysis. A total of 525 participants were thus included in the analytic sample for calculation of cognitive trajectories for the first sensitivity analysis (Figure S1). As with the main analysis, rates of decline were fastest for ADNC + LBD and ADNC + LATE-NC for overall cognition, attention, language, and executive function, with lesser variations among the neuropathologic categories for episodic memory (Table S1). The effects of ADNC + LATE-NC and of ADNC + VBI were similar to what was found in the main analysis. However, the effect of ADNC + LBD was weaker than in the main analysis, with only 2 domains (attention, executive function) significantly worse than ADNC alone (in comparison to 4 domains in the main analysis–overall cognition, attention, language, executive function). This finding likely is related to the inclusion of non-neocortical stages of LBD in the sensitivity analysis.

For the second sensitivity analysis (using broader definitions, plus atherosclerosis or arteriolosclerosis), the sample size for all participants decreased slightly further to 804 due to missing values for atherosclerosis or arteriolosclerosis (n = 3). The number of people who had multiple neuropathologic combinations not included in the neuropsychological analysis rose to 398. A total of 406 participants were thus included in the analytic sample for calculation of cognitive trajectories for the second sensitivity analysis (Figure S2). As with the main analysis and the first sensitivity analysis, rates of decline were fastest for ADNC + LBD and ADNC + LATE-NC for most domains. ADNC + VBI showed more effect than in the main analysis and the first sensitivity analysis, with worse decline compared to ADNC alone for 2 domains (attention, executive function). Nonetheless, it is still < that of ADNC + LBD.

In summary, there were some differences in the effects of ADNC + LBD and ADNC + LATE-NC among the 3 analyses (main and 2 sensitivity). However, in all 3 analyses ADNC + VBI had minimal effects and less effects than the other combinations. In addition, in all 3 analyses, no interactions between any 2 neuropathologic entities were commensurate with a synergistic (worse than additive) effect.

In the final exploratory analysis, we assessed the significance of differences in the 5 cognitive domains for the individual neuropathologies alone (LBD, LATE-NC, VBI) versus in combination with ADNC (LBD + ADNC; VBI + ADNC; LATE-NC + ADNC). This analysis was similar to the main analysis but compared the effects of ADNC + other neuropathologies to the effect of those individual neuropathologies alone rather than to ADNC alone as in the main analysis. In both the exploratory analysis and the main analysis, 9 of 15 possible combinations (3 neuropathologic comparisons for 5 neuropsychological domains) were significantly different for the combined neuropathologies compared to the single neuropathology (Table 3 and Table S3). The following 4 domains were significantly worse (for combined versus single neuropathologies) in the exploratory analysis, but not in the main analysis: overall cognition for VBI; attention for LATE-NC; and language for both VBI and LATE-NC.

## DISCUSSION

This study sought to examine cognitive changes associated with several neuropathologic entities and combinations of those entities. The most notable effects were seen for the combination of ADNC + LBD, which had significantly worse decline than ADNC alone for 4 of the 5 cognitive domains assessed (overall cognition, attention, language, executive function). For all of these 4 domains, the rate of decline was consistent with an additive model. The combination of ADNC + LATE-NC was intermediate with significantly worse decline than ADNC alone for 3 of the 5 domains (overall cognition, episodic memory, executive function). For these 3 domains, the rate of decline was consistent with an additive model for 1 domain (episodic memory) and was < additive (ie, antagonistic) for 2 (overall cognition, executive function). The combination of ADNC + VBI had minimal effect. There was only one domain (episodic memory) for which ADNC + VBI had significantly worse decline than ADNC alone. For this domain, the rate of decline was significantly < anticipated from an additive model (ie, antagonistic). No interactions between any 2 neuropathologic entities were commensurate with a synergistic (worse than additive) effect.

As noted in the Introduction, many studies have examined the effects of combinations of 2 neuropathologies, usually finding that a higher number of neuropathologic entities is associated with worsened cognitive performance. This has been found for combinations of ADNC with each of LBD, microinfarcts, and LATE-NC.^2^^,^^6–16^ This effect has also been observed for overall cognition (as assessed by diagnosis of dementia and by MMSE and CDR, both global and sum of boxes) and for several neuropsychological tests and domains (eg, episodic memory, executive function). A few studies have addressed the question of whether different neuropathologic entities are interacting independently (and hence additively) or whether their effects might be synergistic. Using autopsy data from the Rush Religious Order Study and the Memory and Aging Project, Schneider et al. showed that ADNC and LBD had an additive but not synergistic effect on probability of dementia.^17^ In an in vivo study using imaging to assess the presence of vascular and amyloid neuropathologies, Vemuri et al. showed that these 2 entities had an additive effect on a global cognitive summary score.^16^ In a prior study using NACC autopsy data, Brenowitz et al. looked at 4 neuropsychological test scores for 2 pairings of neuropathologic entities (ADNC + LBD; ADNC + VBI), resulting in 8 possible combinations (of neuropathologic pairings and scores). They found that 6 of the interactions were additive, but 2 were < additive. ADNC + LBD resulted in < expected amount of decline in episodic memory and ADNC + VBI resulted in < expected amount of decline in language.^3^

The current study builds on the findings of the above studies on the quantitative effects of multiple neuropathologic entities. It specifically builds on the findings of Brenowitz et al.^3^ by looking at the same neuropsychological domain scores along with a measure of overall cognitive performance for 3 pairings of neuropathologic entities (ADNC + LBD; ADNC + VBI; ADNC + LATE-NC). In only one domain (episodic memory) was decline worse for ADNC + VBI than for ADNC alone; for this domain, the combination of ADNC + VBI resulted in < additive effects of the 2 neuropathologies. No pairs of neuropathologies acted synergistically, in terms of showing worse decline than expected by an additive model. Thus, none of the studies that have studied at the quantitative effects of neuropathologic combinations have shown synergistic effects. All have shown either additive effects or mixtures of effects that are additive and effects that are antagonistic (less effect on cognition than would be expected if the 2 neuropathologic entities were acting independently, ie, additively). The biological basis for these findings is uncertain. It may imply that once certain neural pathways are damaged by one of the neuropathologic entities, there is less of the pathway remaining to be further damaged by the other neuropathology.

The presence of VBI with ADNC did not result in significant worsening in cognitive decline compared to ADNC alone in most domains (only episodic memory was worse for ADNC+VBI). This is notable given the strong negative effect of cognition of VBI by itself in the current study and in general in the literature.^9^^,^^16^^,^^27–29^ In contrast, 2 in vivo studies found that there was an additive but not synergistic effect for vascular risk factors and imaging evidence of amyloid beta (ie, positron emission tomography) on risk of cognitive impairment.^28^^,^^30^

Part of the reason for the lack of significant differences between ADNC alone and ADNC + VBI in the current study may be because worsening performance associated with VBI alone was < for most other single neuropathologies for most domains (overall cognition, attention, language, executive function) (Table 3). However, this is not likely to account for the significant differences between observed and additive models for ADNC + VBI for almost all domains, ie, except episodic memory. Interestingly, the current study found that ADNC + VBI had significantly less decline than ADNC alone for attention. This is paradoxical and there is no immediately obvious reason for this finding. Potential reasons for this finding could be residual confounding from variables that were not measured in the database or survivor bias.

Sensitivity analyses were undertaken to look at the effects of different, less restrictive, definitions of the neuropathologic entities. There were modest changes in the effects of LATE-NC and of LBD when added to ADNC when compared to the main analysis. However, in all 3 analyses the addition of VBI to ADNC had minimal effects and less effects than the other combinations. In addition, in all 3 analyses, no interactions between any 2 neuropathologic entities were commensurate with a synergistic (worse than additive) effect.

The current study also provides further data on the distribution of combinations of neuropathologic entities. Boyle et al. found 236 unique combinations of neuropathologic entities and Saltiel et al. found 252 unique combinations.^4^^,^^5^ Both studies examined disaggregated neuropathologic entities (eg, plaques and tangles), as opposed to the current study, which examined the distribution of neuropathologic diagnostic categories. Using these categories, 16 combinations were possible (including participants with none of the 4 neuropathologic entities). The number of participants in each category ranged from 0 (for the 3-way combination of LBD, VBI, and LATE-NC) to 304 (33%) with isolated ADNC (Figure 1). The 33% with isolated ADNC in the current study is higher than the 9% reported by Boyle et al.^4^ However, the 33% with isolated ADNC in the main analysis of the current study was in the scenario with restrictive neuropathologic definitions (eg, 2 or more microinfarcts or gross infarct needed to classify a participant as having VBI). With progressively less restrictive neuropathologic definitions, the percentage of participants with isolated ADNC decreased to 22% (181 out of 807, first sensitivity analysis) and 9% (73 out of 804, secondary sensitivity analysis), commensurate with the 9% reported by Boyle et al.^4^

There were several limitations to this study. First, there may have been selection bias as UDS participants and especially those who consent to autopsy are predominantly white and highly educated, limiting the generalizability of the study. Education and race were adjusted for in the regression models to address this issue. Second, although there were 808 participants, the sample size was limited due to the exclusion of previous versions of the neuropathology forms (prior to version 10), which did not assess TDP-43. Due to small sample sizes, neuropathologic combinations with 3 or more categories, and categories that did not include ADNC could not be analyzed. Third, UDS visits prior to 2015 used the C1 neuropsychological test battery while subsequent initial visits used the C2 battery. Change in the test battery used may have introduced bias. This was mitigated by using z-scores. Fourth, autopsy assessments in this study were based on examination of a representative but limited subset of brain regions. This may underestimate the extent of neuropathologic change especially in unsampled areas. Fifth, we examined the relationship between neuropathologic changes and cognition based on neuropathologic changes at autopsy; these data do not imply causation. Proof of causation would necessitate other study designs, such as experimental models or antemortem longitudinal studies with neuropathologies assessed by in vivo biomarkers. Despite these limitations, the study has several strengths, including longitudinal data from a large number of widely distributed ADRCs, enhancing generalizability. Also, data collection methods, including clinical, neuropsychological test batteries, and autopsy procedures, are standardized across the ADRCs leading to high quality, reliable data.

## Conclusions

In this multicenter autopsy study, most participants had ADNC. This was usually co-existent with other neuropathologic entities, with only a third of all participants having isolated ADNC. The combination of ADNC + LBD showed the most notable effects with significantly worse decline than ADNC alone for 4 of the 5 cognitive domains assessed (overall cognition, attention, language, executive function). For all 4 domains, the rate of decline was consistent with an additive model. The combination of ADNC + LATE-NC was intermediate in its effect, with significantly worse decline than ADNC alone for 3 of the 5 domains (overall cognition, episodic memory, executive function). For these 3 domains, the rate of decline was consistent with an additive model for 1 domain (episodic memory) and was < additive (ie, antagonistic) for 2 (overall cognition, executive function). The combination of ADNC + VBI had minimal effect. There was only one domain (episodic memory) for which ADNC + VBI had significantly worse decline than ADNC alone. For this domain, the rate of decline was significantly < anticipated from an additive model (ie, antagonistic). No combinations showed synergistic (worse than additive) effects. This information will be useful for clinical care, especially for prognostication, when in vivo methods of diagnosing the neuropathologic entities improve. This information will also be useful for enriching clinical trials with participants with more precise definitions of their underlying neuropathologic entities.

## Supplementary Material

nlag033_Supplementary_Data
