# Supplementary material for: Effects of concurrent neuropathologies with Alzheimer disease neuropathologic change on cognitive decline: Minimal impact of vascular brain injury compared with other combinations
Source: J Neuropathol Exp Neurol. 2026 Apr 23;85(8):857–67. doi: 10.1093/jnen/nlag033 (PMC13374727; doi:10.1093/jnen/nlag033)
Supplement: Supplementary file 1 [file nlag033_supplementary_data.docx]

Supplementary Figure S1. Distribution of selected neuropathologic entities using broader definitions

Sample size for all combinations: 807

*Excluded from the analysis for cognitive changes.

Sample size for analytic sample for cognitive changes: 525

Neuropathology definitions: ADNC = low, intermediate, or high NIA-AA Alzheimer’s disease neuropathologic change (ADNC) (ABC score); LBD = Lewy body in any region; VBI = any gross infarct or any microinfarcts; LATE/TDP-43 = distribution of TDP-43 immunoreactive inclusions in amygdala, hippocampus, or neocortex.

Number of participants in each neuropathologic category

|  | N using restrictive definitions | N using broad definitions |
| --- | --- | --- |
| No NP | 117 | 24 |
| ADNC Only | 304 | 181 |
| LBD Only | 14 | 10 |
| VBI Only | 64 | 24 |
| LATE-NC Only | 17 | 1 |
| ADNC + LBD | 48 | 95 |
| ADNC + VBI | 119 | 112 |
| ADNC + LATE-NC | 125 | 78 |
| Total | 808 | 525 |

Supplementary Table S1. Annual change in cognitive domain z scores by neuropathologic entities using broader definitions

|  |  | Estimate (95% CI) | | |
| --- | --- | --- | --- | --- |
| **Cognitive Domains** | **Neuropathologic Entities** | **Annual Change in Z Score** | **Difference between ADNC Only and Combinations** | **Difference Between Additive and Observed Slopes** |
| Overall cognitive performance (MMSE or MoCA) | No NP | -0.03 (-0.12, 0.05) |  |  |
|  | ADNC Only | -0.26 (-0.3, -0.22) |  |  |
|  | LBD Only | -0.20 (-0.36, -0.03) |  |  |
|  | VBI Only | -0.12 (-0.22, -0.03) |  |  |
|  | LATE-NC Only | 0.12 (-0.53, 0.77) |  |  |
|  | ADNC + LBD | -0.30 (-0.37, -0.24) | -0.05 (-0.12, 0.02) | 0.15 (-0.03, 0.32) |
|  | ADNC + VBI | -0.26 (-0.31, -0.22) | -0.01 (-0.06, 0.05) | **0.11 (0.01, 0.22)** |
|  | ADNC + LATE-NC | -0.38 (-0.43, -0.32) | **-0.12 (-0.19, -0.06)** | -0.25 (-0.90, 0.41) |
| Episodic memory | No NP | 0.01 (-0.02, 0.04) |  |  |
|  | ADNC Only | -0.02 (-0.04, -0.01) |  |  |
|  | LBD Only | -0.06 (-0.12, -0.01) |  |  |
|  | VBI Only | -0.04 (-0.07, -0.00) |  |  |
|  | LATE-NC Only | 0.19 (-0.04, 0.42) |  |  |
|  | ADNC + LBD | -0.03 (-0.06, -0.01) | -0.01 (-0.04, 0.02) | 0.05 (-0.01, 0.12) |
|  | ADNC + VBI | -0.04 (-0.06, -0.03) | -0.02 (-0.04, 0.002) | 0.02 (-0.02, 0.05) |
|  | ADNC + LATE-NC | -0.07 (-0.09, -0.05) | **-0.04 (-0.07, -0.02)** | -0.24 (-0.47, -0.01) |
| Attention / working memory | No NP | 0.02 (-0.01, 0.05) |  |  |
|  | ADNC Only | -0.05 (-0.06, -0.04) |  |  |
|  | LBD Only | -0.04 (-0.10, 0.01) |  |  |
|  | VBI Only | -0.04 (-0.06, -0.01) |  |  |
|  | LATE-NC Only | 0.04 (-0.16, 0.24) |  |  |
|  | ADNC + LBD | -0.08 (-0.10, -0.06) | **-0.03 (-0.05, -0.003)** | 0.02 (-0.04, 0.08) |
|  | ADNC + VBI | -0.04 (-0.06, -0.03) | 0.01 (-0.01, 0.02) | **0.04 (0.01, 0.07)** |
|  | ADNC + LATE-NC | -0.09 (-0.10, -0.07) | **-0.04 (-0.06, -0.02)** | -0.08 (-0.28, 0.13) |
| Semantic memory / language | No NP | -0.00 (-0.03, 0.03) |  |  |
|  | ADNC Only | -0.11 (-0.12, -0.09) |  |  |
|  | LBD Only | -0.09 (-0.15, -0.04) |  |  |
|  | VBI Only | -0.05 (-0.09, -0.02) |  |  |
|  | LATE-NC Only | 0.13 (-0.09, 0.34) |  |  |
|  | ADNC + LBD | -0.12 (-0.14, -0.10) | -0.01 (-0.03, 0.02) | **0.08 (0.02, 0.14)** |
|  | ADNC + VBI | -0.09 (-0.11, -0.08) | 0.01 (-0.004, 0.03) | **0.07 (0.03, 0.11)** |
|  | ADNC + LATE-NC | -0.12 (-0.14, -0.10) | -0.01 (-0.03, 0.01) | -0.14 (-0.36, 0.08) |
| Executive function | No NP | -0.04 (-0.08, -0.00) |  |  |
|  | ADNC Only | -0.12 (-0.14, -0.10) |  |  |
|  | LBD Only | -0.15 (-0.25, -0.05) |  |  |
|  | VBI Only | -0.10 (-0.14, -0.05) |  |  |
|  | LATE-NC Only | -0.13 (-0.42, 0.15) |  |  |
|  | ADNC + LBD | -0.18 (-0.21, -0.14) | **-0.06 (-0.09, -0.02)** | 0.09 (-0.01, 0.20) |
|  | ADNC + VBI | -0.13 (-0.15, -0.10) | -0.01 (-0.04, 0.02) | **0.09 (0.03, 0.14)** |
|  | ADNC + LATE-NC | -0.16 (-0.18, -0.13) | **-0.04 (-0.07, -0.01)** | 0.09 (-0.20, 0.38) |

Abbreviations: ADNC = Alzheimer disease neuropathologic change; LATE-NC = limbic predominant, age related, TDP-43 encephalopathy neuropathologic change; LBD = Lewy body disease; NP = neuropathology; VBI = vascular brain injury. Each model was adjusted for sex, education, age at death, race, neuropsychological battery form version (C1 or C2), and global CDR® score at baseline.

Supplementary Figure S2. Distribution of selected neuropathologic entities using broader definitions and including atherosclerosis and arteriolosclerosis in the definition of vascular brain injury

Sample size for all combinations: 804

*Excluded from the analysis for cognitive changes.

Sample size for analytic sample for cognitive changes: 406

Neuropathology definitions: ADNC = low, intermediate, or high NIA-AA Alzheimer’s disease neuropathologic change (ADNC) (ABC score); LBD = Lewy body in any region; VBI = any gross infarct or microinfarcts, or moderate to severe atherosclerosis or arteriolosclerosis; LATE/TDP-43 = distribution of TDP-43 immunoreactive inclusions in amygdala, hippocampus, or neocortex.

Number of participants in each neuropathologic category

|  | N using restrictive definitions | N using broad definitions | N using broad definitions including atherosclerosis and arteriolosclerosis for VBI |
| --- | --- | --- | --- |
| No NP | 117 | 24 | 11 |
| ADNC Only | 304 | 181 | 73 |
| LBD Only | 14 | 10 | 6 |
| VBI Only | 64 | 24 | 37 |
| LATE-NC Only | 17 | 1 | 0 |
| ADNC + LBD | 48 | 95 | 37 |
| ADNC + VBI | 119 | 112 | 219 |
| ADNC + LATE-NC | 125 | 78 | 23 |
| Total | 808 | 525 | 406 |

Supplementary Table S2. Annual change in cognitive domain z scores by neuropathologic entities using broader definitions and including atherosclerosis and arteriolosclerosis in the definition of vascular brain injury

|  |  | Estimate (95% CI) | | |
| --- | --- | --- | --- | --- |
| **Cognitive Domains** | **Neuropathologic Entities** | **Annual Change in Z Score** | **Difference between ADNC Only and Combinations** | **Difference Between Additive and Observed Slopes** |
| Overall cognitive performance (MMSE or MoCA) | No NP | -0.02 (-0.14, 0.10) |  |  |
|  | ADNC Only | -0.22 (-0.28, -0.15) |  |  |
|  | LBD Only | -0.04 (-0.27, 0.18) |  |  |
|  | VBI Only | -0.08 (-0.15, -0.01) |  |  |
|  | LATE-NC Only | NA |  |  |
|  | ADNC + LBD | -0.27 (-0.37, -0.17) | -0.05 (-0.17, 0.06) | -0.01 (-0.26, 0.24) |
|  | ADNC + VBI | -0.26 (-0.30, -0.23) | -0.05 (-0.11, 0.02) | 0.03 (-0.06, 0.13) |
|  | ADNC + LATE-NC | -0.33 (-0.43, -0.23) | -0.12 (-0.23, 0.003) | NA |
| Episodic memory | No NP | 0.03 (-0.01, 0.07) |  |  |
|  | ADNC Only | -0.03 (-0.05, -0.002) |  |  |
|  | LBD Only | -0.10 (-0.18, -0.02) |  |  |
|  | VBI Only | -0.02 (-0.05, 0.001) |  |  |
|  | LATE-NC Only | NA |  |  |
|  | ADNC + LBD | -0.06 (-0.09, -0.02) | -0.03 (-0.08, 0.02) | 0.07 (-0.02, 0.16) |
|  | ADNC + VBI | -0.04 (-0.05, -0.03) | -0.01 (-0.04, 0.01) | 0.01 (-0.02, 0.05) |
|  | ADNC + LATE-NC | -0.11 (-0.15, -0.07) | **-0.08 (-0.13, -0.04)** | NA |
| Attention / working memory | No NP | 0.03 (-0.01, 0.06) |  |  |
|  | ADNC Only | -0.02 (-0.04, 0.002) |  |  |
|  | LBD Only | -0.03 (-0.10, 0.03) |  |  |
|  | VBI Only | -0.02 (-0.04, 0.003) |  |  |
|  | LATE-NC Only | NA |  |  |
|  | ADNC + LBD | -0.09 (-0.13, -0.06) | **-0.07 (-0.11, -0.04)** | -0.04 (-0.11, 0.03) |
|  | ADNC + VBI | -0.05 (-0.06, -0.04) | **-0.03 (-0.05, -0.01)** | -0.01 (-0.04, 0.02) |
|  | ADNC + LATE-NC | -0.05 (-0.08, -0.02) | -0.03 (-0.07, 0.01) | NA |
| Semantic memory / language | No NP | 0.03 (-0.01, 0.07) |  |  |
|  | ADNC Only | -0.09 (-0.11, -0.07) |  |  |
|  | LBD Only | -0.11 (-0.19, -0.04) |  |  |
|  | VBI Only | -0.04 (-0.07, -0.02) |  |  |
|  | LATE-NC Only | NA |  |  |
|  | ADNC + LBD | -0.13 (-0.17, -0.10) | **-0.04 (-0.09, -0.003)** | 0.07 (-0.01, 0.15) |
|  | ADNC + VBI | -0.11 (-0.12, -0.09) | -0.01 (-0.04, 0.01) | 0.03 (-0.01, 0.06) |
|  | ADNC + LATE-NC | -0.11 (-0.15, -0.08) | -0.02 (-0.06, 0.02) | NA |
| Executive function | No NP | -0.03 (-0.08, 0.02) |  |  |
|  | ADNC Only | -0.09 (-0.12, -0.06) |  |  |
|  | LBD Only | -0.14 (-0.28, -0.01) |  |  |
|  | VBI Only | -0.08 (-0.11, -0.04) |  |  |
|  | LATE-NC Only | NA |  |  |
|  | ADNC + LBD | -0.24 (-0.29, -0.19) | **-0.15 (-0.21, -0.10)** | -0.01 (-0.15, 0.14) |
|  | ADNC + VBI | -0.13 (-0.14, -0.11) | **-0.04 (-0.07, -0.01)** | 0.04 (-0.01, 0.09) |
|  | ADNC + LATE-NC | -0.19 (-0.24, -0.14) | **-0.10 (-0.16, -0.04)** | NA |

Abbreviations: ADNC = Alzheimer disease neuropathologic change; LATE-NC = limbic predominant, age related, TDP-43 encephalopathy neuropathologic change; LBD = Lewy body disease; NP = neuropathology; VBI = vascular brain injury. Each model was adjusted for sex, education, age at death, race, neuropsychological battery form version (C1 or C2), and global CDR® score at baseline.

Supplementary Table S3. Differences in annual change in cognitive domain z scores between single neuropathology and their corresponding combinations with ADNC

| **Cognitive Domains** | **Neuropathologic Entities** | **Estimate (95% CI)** |
| --- | --- | --- |
| Overall cognitive performance (MMSE or MoCA) | LBD vs LBD + ADNC | **-0.34 (-0.52, -0.16)** |
|  | VBI vs VBI + ADNC | **-0.10 (-0.17, -0.02)** |
|  | LATE-NC vs LATE-NC + ADNC | **-0.23 (-0.35, -0.10)** |
| Episodic memory | LBD vs LBD + ADNC | 0.02 (-0.04, 0.08) |
|  | VBI vs VBI + ADNC | -0.01 (-0.04, 0.01) |
|  | LATE-NC vs LATE-NC + ADNC | -0.04 (-0.08, 0.00) |
| Attention / working memory | LBD vs LBD + ADNC | **-0.09 (-0.14, -0.03)** |
|  | VBI vs VBI + ADNC | -0.03 (-0.05, 0.00) |
|  | LATE-NC vs LATE-NC + ADNC | **-0.06 (-0.10, -0.02)** |
| Semantic memory / language | LBD vs LBD + ADNC | **-0.13 (-0.19, -0.06)** |
|  | VBI vs VBI + ADNC | **-0.06 (-0.09, -0.04)** |
|  | LATE-NC vs LATE-NC + ADNC | **-0.09 (-0.13, -0.05)** |
| Executive function | LBD vs LBD + ADNC | **-0.18 (-0.29, -0.08)** |
|  | VBI vs VBI + ADNC | -0.02 (-0.05, 0.02) |
|  | LATE-NC vs LATE-NC + ADNC | -0.02 (-0.08, 0.03) |

Abbreviations: ADNC = Alzheimer disease neuropathologic change; LATE-NC = limbic predominant, age related, TDP-43 encephalopathy; LBD = Lewy body disease; NP = neuropathology; VBI = vascular brain injury. Each model was adjusted for sex, education, age at death, race, neuropsychological battery form version (C1 or C2), and global CDR® score at baseline.
